# Analysis of Variation Between Diagnosis at Admission vs Discharge and Clinical Outcomes Among Adults With Possible Bacteremia

**DOI:** 10.1001/jamanetworkopen.2022.18172

**Published:** 2022-06-23

**Authors:** Emma Dregmans, Anna G. Kaal, Soufian Meziyerh, Nikki E. Kolfschoten, Maarten O. van Aken, Emile F. Schippers, Ewout W. Steyerberg, Cees van Nieuwkoop

**Affiliations:** 1 Department of Internal Medicine, Haga Teaching Hospital, The Hague, the Netherlands; 2 Department of Internal Medicine, Leiden University Medical Center, Leiden, the Netherlands; 3 Department of Emergency Medicine, Haga Teaching Hospital, The Hague, the Netherlands; 4 Department of Public Health and Primary Care, Leiden University Medical Center, Leiden, the Netherlands; 5 Department of Biomedical Data Sciences, Leiden University Medical Center, Leiden, the Netherlands

## Abstract

**Importance:**

Misdiagnosis of infection is among the most commonly made diagnostic errors and is associated with increased morbidity and mortality. Little is known about how often misdiagnosed site of infection occurs and its association with clinical outcomes.

**Objectives:**

To evaluate the discrepancy between admission and discharge site of infection diagnoses among patients with suspected bacteremia, to explore factors associated with discrepant diagnoses, and to evaluate the association with clinical outcomes.

**Design, Setting, and Participants:**

This cohort study used electronic records of 1477 adult patients who were admitted to the hospital for suspected bacteremia from April 1, 2019, to May 31, 2020, and who had blood cultures taken at the emergency department at Haga Teaching Hospital, The Hague, the Netherlands. Suspected infection sites were classified into 8 categories at admission and discharge. Misdiagnosed site was defined as a discrepancy between the suspected site of infection at admission and at discharge.

**Main Outcomes and Measures:**

Clinical outcomes were 30-day mortality, intensive care unit admission, length of hospital stay, and antibiotic use, analyzed with logistic and linear regression. Risk factors for misdiagnosed site were determined using regression analysis.

**Results:**

A total of 1477 patients (820 [55.5%] male; median [IQR] age, 68 [56-78] years) were analyzed. The rate of misdiagnosed site of infection was 11.6% (171 of 1477); 3.1% of all patients (46 of 1477) ultimately had no infection. No association was found between misdiagnosis and 30-day mortality (adjusted odds ratio [aOR], 0.8; 95% CI, 0.3-1.9; *P* = .60), intensive care unit admission (aOR, 1.3; 95% CI, 0.6-3.0; *P* = .54), and hospital length of stay (adjusted increase of stay, 15.5%; 95% CI, −3.1% to 37.7%; *P* = .11). Misdiagnosed site was associated with receiving broad-spectrum antibiotics (aOR, 4.0; 95% CI, 1.8-8.8; *P* < .001). Older age, dementia, a positive urine sediment test result without urinary symptoms, and suspicion of an intravascular, central nervous system, or bone and joint infection were risk factors for misdiagnosed site of infection.

**Conclusions and Relevance:**

In this cohort study, misdiagnosed site of infection occurred in 1 of 9 patients and was not associated with worse short-term clinical outcomes. Clinicians should be aware of risk factors associated with misdiagnosed site of infection and potential inappropriate antibiotic use.

## Key Points

**Question:**

What is the incidence of incorrect identification of site of infection at time of admission, and is this misidentification associated with clinical outcomes?

**Findings:**

In this cohort study of 1477 patients admitted for suspected bacterial infection, misdiagnosed site of infection occurred in 12% of hospitalized patients. No association was found between misdiagnosed site of infection and 30-day mortality, intensive care unit admission, and length of stay, which might be explained by the use of broad-spectrum antibiotics.

**Meaning:**

These findings suggest that misdiagnosed site of infection occurs in every 1 of 9 hospitalized patients, but it is not associated with worse clinical outcomes.

## Teaser

This cohort study of emergency department patients with suspected bacterial infection examines the rate of misdiagnosed site of infection and the association of misdiagnosis with clinical outcomes.

## Introduction

Increasing attention is being paid to the prevention of diagnostic errors, including misdiagnoses, because these errors have been associated with inappropriate treatment, harm to patients, and increasing costs.^1,2,3^ Little is known about the incidence of misdiagnoses in hospitals and their consequences.^4^ The common overall estimated percentage of diagnostic error is 10% to 15%, but great variation exists among studies because of a lack of unified terminology and different settings of studies.^4,5,6,7,8,9^ In this respect, emergency departments (EDs) are known to have higher rates of misdiagnoses because of the complex decision-making process, limited available clinical information, high workload, and time constraints.^2,9,10,11^

A study^12^ among tertiary-level EDs in Japan on the misdiagnosis of infections found that 11.6% (113 of 974) of ED patients had a misdiagnosis, with urinary tract infection being the most common misdiagnosis. Misdiagnosed infections may lead to morbidity and mortality, as well as to inappropriate diagnostic testing and antimicrobial treatment, and thus contribute to increasing antimicrobial resistance.^4,11,13,14^ Nonspecific symptoms related to infections make it challenging to establish the correct diagnosis. Fever, for example, is a symptom of low specificity and may indicate a self-limiting infection as well as severe sepsis.^15,16^

Identifying risk factors for misdiagnosis might be helpful to improve diagnostic accuracy. Previous studies^9,10^ have primarily focused on assessing the type of error in the diagnostic process and on the predictive role of context (eg, noise) and work experience in misdiagnoses. Data on patient factors associated with misdiagnoses are lacking.

We recently performed an observational study^17^ of all adults presenting to the ED in a large teaching hospital in the Netherlands from whom a blood culture was taken because of suspected bacteremia. The aims of the current substudy were to evaluate the rate of misdiagnosed site of infection in ED patients with suspected bacteremia, to examine the association of misdiagnosis with clinical outcomes, and to identify risk factors associated with misdiagnosis.

## Methods

### Study Design and Setting

The current study is a substudy of PredictED (Predicting Microbiological and Clinical Outcomes of Patients With Suspected Bacteremia Presenting at Emergency Department: A Prospective Observational Study),^17^ a single-center observational cohort study that was conducted at the ED of the Haga Teaching Hospital in The Hague, the Netherlands, a large hospital with approximately 600 beds and 50 000 ED visits per year. A total of 2333 adult patients who presented at the ED of Haga Teaching Hospital and had a blood culture taken from April 1, 2019, to May 31, 2020, were included in the study. For this substudy, patients were excluded if they were not hospitalized or transferred to another hospital during their admission, because in such cases the final diagnosis was not available. In addition, patients who were not diagnosed with any infection by the ED physician or patients with an unidentified site of infection at the ED were excluded. PredictED was approved by the Haga Teaching Hospital Medical Ethics Committee and the Haga Teaching Hospital Institutional Scientific Review Board. The need for informed consent was waived because this is an observational quality control study without any effect on patients’ medical management. The study followed the Strengthening the Reporting of Observational Studies in Epidemiology (STROBE) reporting guideline.

### Data Collection

Demographic characteristics and clinical and biochemical findings were collected from the patient electronic records by 2 investigators (E.D. and A.G.K.). Site of infection was obtained by reviewing the patient record, including the hospital discharge letter. A Cohen κ score was used to assess interrater agreement between the investigators (E.D. and A.G.K.). In case of disagreement, a third internal medicine physician was consulted (S.M.). The primary outcome measures were the rate of misdiagnosed infection, 30-day mortality, and intensive care unit (ICU) admission. Secondary outcomes included the length of hospital stay and the administration of broad-spectrum antibiotics.

### Data Definitions

Suspected infection sites were classified into 8 categories: respiratory, urogenital, skin and soft tissue, central nervous system (CNS), abdominal, intravascular, bone and joint, and other infection. The diagnosis at discharge was classified into identical categories as well as unidentified site of infection and no infection. Misdiagnosed site was defined as a discrepancy between the suspected site of infection at admission and at discharge. In case of a possible second infection, a misdiagnosis was defined as a discrepancy between the infection site listed as the reason for hospitalization at admission and at discharge. The quick Sepsis Related Organ Failure Assessment (qSOFA) score was used as a measure for disease severity, and the Charlson Comorbidity Index was used to measure the burden of comorbidity.^18,19^ Urinary symptoms were defined as occurrence of any Loeb criteria (dysuria, suprapubic pain/tenderness, frequency, or urgency).^20^ A positive urine sediment test result indicated a positive nitrite test result or more than 5 white blood cells per high-power field. Broad-spectrum antibiotics were defined as intravenous second- or third-generation cephalosporin with or without an aminoglycoside.^21^ Bacteremia was present when a blood culture, taken within 48 hours after presentation at the ED, showed bacterial growth and was not deemed a contaminant. Coagulase-negative staphylococci, *Bacillus* species, *Corynebacterium* species, *Cutibacterium* species, *Viridans* group streptococci, and *Lactobacillus* species were considered contaminants unless associated with an intravascular catheter or device, suspected endocarditis, or the presence of multiple positive blood culture results with the same bacterium.

### Statistical Analysis

Descriptive statistics were presented as numbers and percentages for categorical variables. All continuous variables were expressed in medians and IQRs. The χ^2^ test or Fisher exact test for categorical variables was used. Continuous variables were tested with the Mann-Whitney *U* test. A 2-sided *P* < .05 was considered statistically significant. Rates of misdiagnosis were calculated for each site of infection.

Univariate and multivariable regression analyses were performed to investigate the association of misdiagnoses with outcomes. The binary dependent variables were 30-day mortality, ICU admission, and administration of broad-spectrum antibiotics, which were analyzed with logistic regression models. A linear model was used for the length of hospital stay with a logarithmic transformation because of skewed data. Multivariable analyses were performed to correct for age, Charlson Comorbidity Index, qSOFA score, and site of infection at discharge. A sensitivity analysis investigated the association of misdiagnosis with clinical outcomes when patients with no infectious disease at discharge were excluded.

To identify variables associated with misdiagnosed sites, a multivariable logistic regression model was built using the LASSO (least absolute shrinkage and selection operator) method.^22^ Eighteen candidate predictor parameters were considered for inclusion based on a sample size of 1900 participants, an outcome proportion of 15%, and an expected shrinkage of 10%. These variables were age, sex, dementia, use of immunosuppressants, recent use of antibiotics, altered mental status, systolic blood pressure, breathing frequency, C-reactive protein level, presence of Loeb criteria, positive urine sediment test result without Loeb criteria, and suspected site of infection at the ED (8 site categories).^20^ Missing data (1.4%) were assumed to occur at random and imputed 10 times using chained equations.^23^ The variables that contained missing values were altered mental status, systolic blood pressure, breathing frequency, C-reactive protein level, and positive urine sediment test result without Loeb criteria. The 10 resulting imputed data sets were stacked into a single large data set and then incorporated into 1 model through weights. Because of the many different diagnoses within the group labeled as “other infection,” this category was excluded in the LASSO analysis. Internal validation was conducted with a 200-bootstrap resampling procedure, where the LASSO procedure was repeated in each bootstrap sample to estimate the optimism-corrected performance.^24^ Associations were presented as regression coefficients for linear regression and odds ratios (ORs) for binary outcomes. With the use of the LASSO method, model selection through shrinkage is achieved to reduce overfitting. Because of shrinkage, CIs cannot be determined for the final set of ORs, in contrast to the full model, including all candidate variables. Discrimination of the model was assessed with the concordance statistic (C statistic), an equivalent to the area under the receiver operating characteristic curve. Statistical analyses were performed using R software, version 1.3.1073 (R Foundation for Statistical Computing) and IBM SPSS Statistics, version 26 (IBM Inc).

## Results

Between April 2019 and May 2020, a total of 2333 patients presented at the ED of Haga Teaching Hospital and had a blood culture taken. Of these, 1477 patients were admitted to the hospital with a suspected infection (eFigure in the Supplement). The median age was 68 years (IQR, 56-78 years), 657 (44.5%) were female, and 820 (55.5%) were male. A total of 171 admitted patients (11.6%) had a misdiagnosed site of infection (Table 1). The Cohen κ for interrater agreement of site of infection was 0.87.

**Table 1.** Characteristics of 1477 Patients With Misdiagnosed and Correctly Diagnosed Site of Infection^a^

Characteristic	Misdiagnosed (n = 171)	Correctly diagnosed (n = 1306)	*P* value
Demographic characteristics and medical history			
Age, median (IQR), y	72 (64-80)	68 (55-78)	<.001
Sex			
Male	89 (52)	731 (56)	.33
Female	82 (48)	575 (44)	
Nursing home resident	18 (10.5)	143 (10.9)	.87
History of cardiovascular disease	96 (56.1)	544 (41.7)	<.001
History of pulmonary disease	34 (19.9)	392 (30.0)	<.001
History of kidney disease	34 (19.9)	151 (11.6)	.002
Dementia	14 (8.2)	50 (3.8)	.008
Charlson Comorbidity Index, median (IQR)	4 (2-6)	3 (1-5)	.003
Vital signs			
Initial SBP, median (IQR), mm Hg	128 (110-150)	129 (112-148)	.49
Initial HR, median (IQR), beats/min	98 (84-115)	102 (89-115)	.16
Initial BF, median (IQR), breaths/min	20 (16-25)	20 (16-26)	.08
Altered mental status	28 (16.8)	105 (8.9)	.001
Initial body temperature, median (IQR), °C	38.5 (37.8-39.3)	38.6 (37.9-39.2)	.40
qSOFA score			
0	67 (43.5)	496 (44.7)	.10
1	59 (38.3)	475 (42.8)	
2	23 (14.9)	118 (10.6)	
3	5 (3.2)	21 (1.9)	
Laboratory results			
CRP, median (IQR), mg/dL	10.0 (4.0-18.3)	9.5 (3.5-19.7)	.95
WBCs, median (IQR), /μL	11 300 (8600-16 100)	12 100 (8400-16 800)	.44
Positive urine sediment test result^b^	74 (43.3)	429 (32.8)	.007
Bacteremia^c^	42 (24.6)	218 (16.7)	.01

Abbreviations: BF, breathing frequency; CRP, C-reactive protein; HR, heart rate; qSOFA, quick Sequential Organ Failure Assessment; SBP, systolic blood pressure; WBCs, white blood cells.

SI conversion factors: To convert CRP to milligrams per liter, multiply by 10; WBCs to ×10^9^/L, multiply by 0.001.

^a^ Data are presented as number (percentage) of patients unless otherwise indicated. Systolic blood pressure data are missing in 7 patients, HR data in 4 patients, BF data in 93 patients, altered mental status data in 131 patients, CRP data in 1 patient, WBC count data in 2 patients, data on leukocytes in urine sediment in 6 patients, and qSOFA data in 213 patients.

^b^ Positive urine sediment test result indicates nitrite positive or more than 5 WBCs per high-power field.

^c^ Present when within 48 hours after presentation at the emergency department a blood culture yielded a pathogen typical for the infection site.

### Patient Characteristics 

Patients with misdiagnosed site of infection were older (median age, 72 [IQR, 64-80] vs 68 [IQR, 55-78] years; *P* < .001) (Table 1); more likely to have a history of cardiovascular disease (96 [56.1%] vs 544 [41.7%]; *P* < .001), kidney disease (34 [19.9%] vs 151 [11.6%]; *P* = .002), or dementia (14 [8.2%] vs 50 [3.8%]; *P* = .008); and had a higher Charlson Comorbidity Index (median, 4 [IQR, 1-6] vs 3 [IQR, 2-5]; *P* = .003). Initial vital signs were similar across groups. More patients with misdiagnosed site of infection had altered mental status (28 [16.8%] vs 105 [8.9%]; *P* = .001), a positive urine sediment test result (74 [43.3%] vs 429 [32.8%]; *P* = .007), and bacteremia (42 [24.6%] vs 218 [16.7%]).

### Rate of Misdiagnoses

The most common sites of infections as diagnosed at admission were respiratory (657 of 1477 [44.5%]) and urogenital (336 of 1477 [22.7%]). Bone and joint (12 of 38 [31.6%]), CNS (8 of 21 [38.1%]), and intravascular (14 of 30 [46.7%]) infections had the highest rates of misdiagnosed site (Figure). The rate of misdiagnoses per initial sites of infection are outlined in the Figure. Thirteen patients had a possible second infection at admission, 5 at both admission and discharge, and 7 at discharge only. Of all 13 patients with a possible second site of infection, 8 (61.5%) were misdiagnosed. Among all 1477 patients, 46 (3.1%) were ultimately discharged with a diagnosis of no infection (Table 2).

**Figure.**
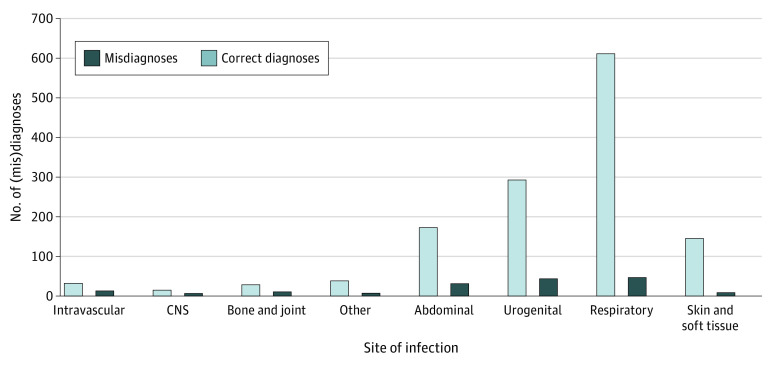
Misdiagnoses per Site of Infection CNS indicates central nervous system.

**Table 2.** Rate of Misdiagnosis by Site of Infection in the 1477 Study Patients

Site of infection at ED	Misdiagnosed sites (n = 171), No./total No. (%)	No. of infections by site of infection at discharge									
		Respiratory	Urogenital	Abdominal	Skin and soft tissue	Bone and joint	Intravascular	CNS	Other^a^	No infection	Unidentified
Respiratory	46/657 (7.0)	611	11	6	3	0	0	1	3	15^b^	7
Urogenital	43/336 (12.8)	9	293	8	3	1	4	2	3	5^c^	8
Abdominal	30/203 (14.8)	4	10	173	0	0	3	0	1	8^d^	4
Skin and soft tissue	10/145 (6.9)	1	1	1	135	4	2	0	0	1^e^	0
Bone and joint	12/38 (31.6)	0	1	0	0	26	0	0	1	10^f^	0
Intravascular	14/30 (46.7)	1	3	2	1	0	16	0	0	4^g^	3
CNS	8/21 (38.1)	1	2	0	0	1	1	13	2	1^h^	0
Other^a^	8/47 (17.0)	3	1	0	1	0	0	0	39	2^i^	1

Abbreviations: CNS, central nervous system; ED, emergency department.

^a^ Other sites of infection (eg, ear, nose, and throat) and viral infection.

^b^ Pulmonary embolism (n = 2), congestive heart failure (n = 2), carcinoma (n = 2), chemical pneumonia (n = 1), neutropenic fever after chemotherapy (n = 1), fever after radiation therapy (n = 1), absorption fever attributable to hematoma (n = 1), pleural effusion (n = 1), nitrofurantoin-induced acute pulmonary toxicity (n = 1), lytic rib lesion (n = 1), symptoms after thoracic endovascular aortic repair (n = 1), and unexplained syncope (n = 1).

^c^ Terminal heart failure (n = 1), metastatic malignancy (n = 1), delirium (n = 1), terminal kidney insufficiency (n = 1), and dehydration (n = 1).

^d^ Hepatic encephalopathy (n = 1), silent jaundice (n = 1), hepatic lymphoma (n = 1), inflammatory bowel disease (n = 1), fever after immunotherapy (n = 1), benign paroxysmal positional vertigo (n = 1), ileus (n = 1), and ischemic colitis (n = 1).

^e^ Lymphoma (n = 1).

^f^ Arthrosis (n = 2), muscle complaints (n = 2), tophaceous gout (n = 2), calcium pyrophosphate deposition arthritis (n = 2), psoriatic arthritis (n = 1), and osteoarthritis (n = 1).

^g^ Disseminated intravascular coagulation in metastatic malignancy (n = 1), portal vein thrombosis (n = 1), mitral valve vegetation without clinical symptoms (n = 1), and suspected endocarditis with negative blood culture.

^h^ Hepatic encephalopathy (n = 1).

^i^ Febrile neutropenia in acute myeloblastic leukemia (n = 1) and metastatic malignant neoplasm (n = 1).

### Association of Misdiagnosis With Clinical Outcomes

Of 171 patients with a misdiagnosed site of infection, 18 (10.5%) died within 30 days of presentation (Table 3); in patients with correctly diagnosed sites, the 30-day mortality was 8.5% (111 of 1306). Among patients with misdiagnosed sites, 11 (6.4%) were admitted to the ICU, compared with 89 patients (6.8%) with correctly diagnosed sites. These differences were not statistically significant because a misdiagnosed site of infection was not associated with 30-day mortality (adjusted OR [aOR], 0.8; 95% CI, 0.3-1.9; *P* = .60) or ICU admission (aOR, 1.3; 95% CI, 0.6-3.0; *P* = .53). A sensitivity analysis, excluding patients with no infectious diagnosis at discharge, also showed no association between misdiagnosed site of infection and 30-day mortality (aOR, 0.8; 95% CI, 0.3-1.8; *P* = .55) or ICU admission (aOR, 1.3; 95% CI, 0.6-3.0; *P* = .51). For patients with misdiagnosed sites, the median (IQR) hospital length of stay was 5 days (IQR, 3-9 days), compared with 4 days (IQR, 2-7 days) for patients with correctly diagnosed sites (*P* = .007), a 22.3% (95% CI, 5.6%-41.5%) increase in hospital length of stay; however, when adjusted for confounders, this association disappeared (15.5%; 95% CI, −3.1% to 37.7%; *P* = .11). Most patients (149 of 171 [87.1%] with misdiagnosed sites and 1110 of 1306 [84.9%] with correctly diagnosed sites) received antibiotic therapy. A statistically significantly higher percentage of misdiagnosed patients received broad-spectrum antibiotics compared with patients with a correct diagnosis (86 of 112 [76.8%] vs 532 of 917 [58.0%]; aOR, 4.0; 95% CI, 1.8-8.8; *P* < .001).

**Table 3.** Outcomes of 1477 Patients With Misdiagnosed and Correctly Diagnosed Site of Infection

Outcome	Misdiagnosed site^b^ (n = 171)	Correctly diagnosed site^b^ (n = 1306)	Univariate		Multivariable^a^	
			OR (95% CI)	*P* value	aOR (95% CI)	*P* value
30-d Mortality	18 (10.5)	111 (8.5)	1.3 (0.7 to 2.1)	.38	0.8 (0.3 to 1.9)	.60
ICU admission	11 (6.4)	89 (6.8)	0.9 (0.5 to 1.8)	.85	1.3 (0.6 to 3.0)	.53
Broad-spectrum antibiotic therapy^c^	86/112 (76.8)	532/917 (58.0)	2.4 (1.5 to 3.8)	<.001	4.0 (1.8 to 8.8)	<.001
Hospital length of stay, median (IQR), d^d^	5 (3 to 9)	4 (2 to 7)	22.3 (5.6 to 41.5)^e^	.007	15.5 (−3.1 to 37.7)^e^	.11
Sensitivity analysis^f^						
30-d Mortality	10 (8.0)	111 (8.5)	0.9 (0.5 to 1.8)	.85	0.8 (0.3 to 1.8)	.55
ICU admission	9 (7.2)	89 (6.8)	1.1 (0.5 to 2.2)	.87	1.3 (0.6 to 3.0)	.51

Abbreviations: aOR, adjusted odds ratio; ICU, intensive care unit; OR, odds ratio.

^a^ Adjusted for age, Charlson Comorbidity Index, quick Sequential Organ Failure Assessment, and site of infection at discharge.

^b^ Data are presented as number (percentage) of patients unless otherwise indicated.

^c^ Intravenous second- or third-generation cephalosporin with or without an aminoglycoside administered within 8 hours after a blood culture was taken.

^d^ Excluding patients who died within 30 days.

^e^ Log-linear model in which the exponentiated regression coefficient represents the percentage of change in geometric means when all other variables are held at a fixed value.

^f^ Excluding patients with no infectious disease at discharge.

### Variables Associated With Misdiagnosis

A multivariable logistic regression model was used to assess variables associated with a misdiagnosed site of infection. In Table 4, both the candidate variables and the selected variables are shown with corresponding ORs (regression coefficients can be found in the eTable in the Supplement). Four of 13 variables were selected using the LASSO logistic regression: age, dementia, a positive urine sediment test result without Loeb criteria, and site of infection at ED. Discrimination of the overall model was moderate, with a C statistic of 0.70 (95% CI, 0.68-0.70). The performance remained similar after correcting for optimism (C = 0.69; 95% CI, 0.64-0.74).

**Table 4.** Full Model and Final Determinants of Misdiagnosed Site of Infection

Variables	Full model OR (95% CI)	Reduced model OR^a^^,^^b^
Age (per decade)	1.2 (1.1-1.3)	1.1
Sex (male)	0.9 (0.7-1.3)	Not selected
Use of immunosuppressants	1.1 (0.6-1.8)	Not selected
Use of antibiotics	0.9 (0.6-1.3)	Not selected
Altered mental status	1.0 (0.7-1.7)	Not selected
SBP	1.0 (1.0-1.0)	Not selected
CRP	1.0 (1.0-1.0)	Not selected
BF	1.0 (1.0-1.0)	Not selected
Occurrence of Loeb criteria	1.3 (0.8-2.2)	Not selected
Dementia	1.9 (0.9-3.7)	1.4
Positive urine sediment test result without Loeb criteria^c^^,^^d^	1.4 (0.9-2.1)	1.1
Site of infection at ED		
Abdominal	2.2 (1.3-3.7)	1.4
Bone and joint	6.1 (2.8-13.4)	3.4
CNS	11.0 (4.0-29.7)	5.1
Intravascular	12.0 (5.3-27.2)	6.7
Respiratory	1 [Reference]	1 [Reference]
Urogenital	1.6 (1.0-2.7)	1.2
Skin and soft tissue	1.1 (0.5-2.2)	1.0

Abbreviations: BF, breathing frequency; CNS, central nervous system; CRP, C-reactive protein; ED, emergency department; OR, odds ratio; SBP, systolic blood pressure.

^a^ Model selection is achieved by shrinkage, so the ORs with CIs cannot be determined.

^b^ The tuning parameter (λ) is chosen by cross-validation.^25^ The value of λ that gives the simplest model but also lies within 1 SE of the optimal value of λ was selected.

^c^ The Loeb criteria are dysuria, suprapubic pain or tenderness, frequency, or urgency. A positive urine sediment test result indicates nitrite positive or more than 5 white blood cells per high-power field.

^d^ Occurred in 36 of 163 misdiagnosed patients (22.1%) and 183 of 1267 nonmisdiagnosed patients (14.4%).

## Discussion

This cohort study analyzed misdiagnoses of infection site among ED patients hospitalized for suspected infection. We found a misdiagnosis rate of 11.6% but no association between misdiagnosed site of infection and worse clinical outcomes, including 30-day mortality, need for ICU admission, and length of hospitalization. However, more patients with misdiagnosed sites of infection received broad-spectrum antibiotics. Older age, presence of dementia, a positive urine sediment test result without urinary symptoms, and particular suspected sites of infection (intravascular, CNS, and bone and joint) were the most prominent risk factors associated with misdiagnosed site of infection in our study.

Literature on misdiagnosed site of infection among hospitalized ED patients is scarce. To our knowledge, this is the first study to investigate the association between misdiagnoses and clinical outcomes while simultaneously identifying patient risk factors for misdiagnosed site of infection in this specific population. A Japanese study by Abe et al^12^ investigated the association of misdiagnosed site of infection with clinical outcomes in 974 patients, using a similar definition. However, Abe et al^12^ also included patients with an unidentified site of infection at admission. When these patients were excluded, they found an overall rate of misdiagnoses of 8.1% (76 of 937).

In our study, sites of infection that were less common (eg, intravascular and CNS) were associated with the highest risks for misdiagnosis, which is in line with previous findings.^12^ The higher rate of misdiagnoses in more uncommon sites of infections might be explained by a lack of exposure or more difficulty in identifying infections at less common sites. However, we think that the higher rate of misdiagnoses is also a reflection of the more severe consequences of missing a diagnosis in these categories, which leads physicians to err on the side of caution. For instance, it can be challenging to distinguish septic arthritis from a simple gout. Misdiagnosis of abdominal infections was more often reported in our study than in the study by Abe et al^12^ (30 of 203 [14.8%] vs 7 of 186 [3.8%]). Another study^11^ that evaluated misdiagnoses of pneumonia in the ED found no respiratory site of infection in 26 of 195 patients (13.3%) on discharge from the internal medicine ward, in contrast to 46 of 657 (7.0%) misdiagnosed respiratory infections in our study. We hypothesize that besides differences in study populations, the differences are partly explained by the challenges in this field of research to assess records objectively.

Other studies^2,10^ have shown that diagnostic errors in EDs can result in serious patient harm. A possible explanation for the difference with our study is our focus on patients with infections and suspected bacteremia or sepsis. Most patients with a misdiagnosed site of infection in our study were treated with broad-spectrum antibiotics, following sepsis guidelines. Broad-spectrum antibiotics were likely an effective treatment for various sites of infection, even though an incorrect site was diagnosed. As such, we did not find an association of misdiagnosed infection site with ICU admissions or mortality even though patients with misdiagnosed sites had a higher rate of bacteremia. However, the effects of misdiagnosed site of infection on clinical outcomes may be much broader and more subtle than what we examined in this study. For example, misdiagnosed site might be associated with additional diagnostic testing. Based on our results, we noticed that 20% of misdiagnosed patients received antibiotics unnecessarily, because the final correct diagnosis was no infection. Unnecessary use of antibiotics may lead to adverse effects for individual patients and may contribute to overall increasing antimicrobial resistance, which is considered a large public health threat.^25^ Our unadjusted results showed an association between misdiagnosed site of infection and hospital length of stay, but after adjustment for age, severity of disease, and site of infection at discharge, this association disappeared. When adjusting for these confounders one by one, the association disappeared when corrected for site of infection at discharge. In contrast, Abe et al^12^ demonstrated that patients with a misclassified site of infection at admission had 2.7-fold higher odds of in-hospital mortality. This difference from our results could be explained by differences in study populations, with their cohort having a higher median age, more patients with severe disease as shown by a qSOFA of 2 or greater (38.7% vs 11.3%), and a higher overall mortality (15% vs 8.7%). More research is needed to clarify this discrepancy in the association of misdiagnosed infections with adverse outcomes. We propose that in future research more microbiological outcomes should be evaluated, preferably including long-term outcomes, such as the emergence of antibiotic resistance in patients receiving inappropriate antibiotics.

In this study, we built a model to help physicians identify patients at high risk of a misdiagnosed site of infection using age, dementia, presence of a positive urine sediment test result without Loeb criteria, and the suspected site of infection at ED. The selection of these variables is not unexpected because accurate diagnoses among older adults are complicated by their frequently atypical presentation. Nonspecific symptoms, such as altered mental status or malaise, are more prevalent in older patients presenting to the ED and may further increase diagnostic uncertainty.^26^ Furthermore, patients with dementia may have difficulty expressing their symptoms. Finally, distinguishing real urinary tract infections from asymptomatic bacteriuria can be challenging, as demonstrated by Flokas et al,^27^ who showed that the presence of nonspecific signs and symptoms and overreliance on laboratory tests may explain the excessive misinterpretation of asymptomatic bacteriuria as being a urinary tract infection. The C statistic of 0.69 indicates a moderate performance and hence will probably not directly benefit the clinician. However, we report this as a starting point for future research.

### Strengths and Limitations

A strength of this study is its comprehensiveness, including all adult patients from whom a blood culture was taken at an ED of a large hospital during a 1-year period, reflecting common daily practice. This study also has some limitations. First, this study involves electronic records and may therefore be subject to information bias. Second, the study only looked at differences between sites of infection, but a wrong diagnosis within the same site of infection (eg, diverticulitis and cholecystitis) may also have implications. Third, we have not assessed at what time during a hospital stay the diagnosis has changed and how this might affect the results. Fourth, we do not know whether prescribed antibiotics were appropriate for the actual infection.

## Conclusions

In this cohort study, misdiagnosed site of infection occurred in 1 of 9 patients hospitalized for suspected bacteremia but was not associated with worse short-term clinical outcomes. This finding might be explained by adherence to sepsis guidelines. Risk factors for misdiagnosed site of infection include older age, dementia, a positive urine sediment test result without urinary symptoms, and particular suspected sites of infection (intravascular, CNS, and bone and joint). Additional studies are needed to confirm these risk factors for misdiagnosis of infection at the ED and to further explore the association of misdiagnosed site of infection and adverse outcomes.

## Notes

Supplement. **eTable.** Final Determinants of Misdiagnosis **eFigure.** Flow Diagram of Patients
