# Supplementary material for: Analysis of Variation Between Diagnosis at Admission vs Discharge and Clinical Outcomes Among Adults With Possible Bacteremia
Source: JAMA Netw Open. 2022 Jun 23;5(6):e2218172. doi: 10.1001/jamanetworkopen.2022.18172 (PMC9226997; doi:10.1001/jamanetworkopen.2022.18172)
Supplement: Supplement. — eTable. Final Determinants of Misdiagnosis eFigure. Flow Diagram of Patients [file jamanetwopen-e2218172-s001.pdf]

Supplementary Online Content

Dregmans E, Kaal AG, Meziyerh S, et al. Analysis of variation between diagnosis at admission vs discharge and clinical outcomes among adults with possible bacteremia. *JAMA Netw Open*. 2022;5(6):e2218172. doi:10.1001/jamanetworkopen.2022.18172

eTable. Final Determinants of Misdiagnosis

eFigure. Flow Diagram of Patients

This supplementary material has been provided by the authors to give readers additional information about their work.

**eTable.** Final Determinants of Misdiagnosis[abc]	
Candidate predictors	B (Lasso)
Age (per decade)	0.110
Dementia	0.359
Positive urine sediment with no Loeb criteria[de]	0.139
Site of infection at ED	
Abdominal	0.333
Bone and Joint	1.217
CNS	1.628
Intravascular	1.896
Respiratory	reference
Skin and Soft tissue	0
Urogenital	0.156

[a] Abbreviations: B regression coefficients, CNS central nervous system, ED emergency department, Lasso Least Absolute Shrinkage and Selection Operator.

[b] One-standard error rule was applied to the optimal lambda value, c-statistic (0.70).

[c] The predicted probability of a misdiagnosis was determined by P = 1/[1+ exp - (-3.04 + 0.01 x age (year) + 0.36 x dementia + 0.14 x positive urine sediment with no Loeb criteria + 0.33 x abdominal focus + 1.22 x bone/joint focus + 1.63 x CNS focus + 1.90 x intravascular focus + 0.16 x urogenital focus)].

Instruction: If a predictor is scored positively, the given weight needs to be filled in.

[d] Loeb criteria means dysuria, suprapubic pain/tenderness, frequency or urgency. Positive urine sediment indicates nitrite positive or >5 white blood cells per high-power field.

[e] Occurred in 22.1% (36/163) of the misdiagnosed patients and 14.4% (183/1267) in the correctly diagnosed patients.

© 2022 Dregmans E et al. *JAMA Network Open.*

eFigure. Flow Diagram of Patients

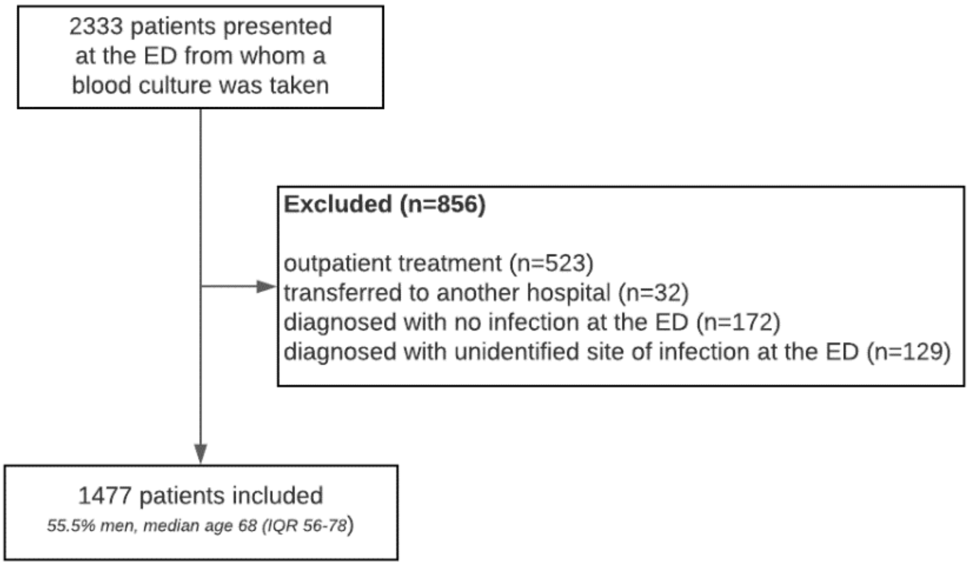

Flow diagram of inclusion.

© 2022 Dregmans E et al. *JAMA Network Open.*
